# PPAR*γ* agonists inhibit growth and expansion of CD133+ brain tumour stem cells

**DOI:** 10.1038/sj.bjc.6604786

**Published:** 2008-11-18

**Authors:** W Chearwae, J J Bright

**Affiliations:** 1 Neuroscience Research Laboratory, Methodist Research Institute at Clarian Health, Indianapolis, IN, USA; 2 Department of Medicine, Indiana University School of Medicine, Indianapolis, IN, USA

**Keywords:** brain tumour stem cells, glioblastoma, PPAR*γ*, anti-cancer drug, Jak-Stat pathway

## Abstract

Brain tumour stem cells (BTSCs) are a small population of cells that has self-renewal, transplantation, multidrug resistance and recurrence properties, thus remain novel therapeutic target for brain tumour. Recent studies have shown that peroxisome proliferator-activated receptor gamma (PPAR*γ*) agonists induce growth arrest and apoptosis in glioblastoma cells, but their effects on BTSCs are largely unknown. In this study, we generated gliospheres with more than 50% CD133+ BTSC by culturing U87MG and T98G human glioblastoma cells with epidermal growth factor (EGF) and basic fibroblast growth factor (bFGF). *In vitro* treatment with PPAR*γ* agonist, 15-Deoxy-Δ^12,14^-Prostaglandin J_2_ (15d-PGJ2) or all-*trans* retinoic acid resulted in a reversible inhibition of gliosphere formation in culture. Peroxisome proliferator-activated receptor gamma agonists inhibited the proliferation and expansion of glioma and gliosphere cells in a dose-dependent manner. Peroxisome proliferator-activated receptor gamma agonists also induced cell cycle arrest and apoptosis in association with the inhibition of EGF/bFGF signalling through Tyk2-Stat3 pathway and expression of PPAR*γ* in gliosphere cells. These findings demonstrate that PPAR*γ* agonists regulate growth and expansion of BTSCs and extend their use to target BTSCs in the treatment of brain tumour.

Brain tumours are the most devastating cancers that present unique challenges to therapy and pose major health problems in the United States and other parts of the world. There are over 100 different types of brain tumours identified in humans that show widely divergent biological and clinical outcomes. Among them, glioblastoma is the most frequent primary malignant brain tumour in adults. Median survival is generally less than 1 year from the time of diagnosis, and even in most favourable situations, patients die within 2 years (Deorah *et al*, 2006). Standard therapy for glioblastoma consists of surgical resection to the extent that is safely feasible, followed by radiotherapy and chemotherapy, which have significant side-effects and limited efficacy (Peacock and Lesser, 2006). Targeted molecular therapies with improved efficacy and reduced toxicity have been developed, but still face many challenges (Kim and Glantz, 2006). Despite recent advances in surgery, radiation, chemotherapy and other molecular therapies, a cure for brain tumours remains elusive. The multidrug resistance and fast recurrence are some of the challenges in combating brain tumours, which warrant further investigation on identifying novel molecular targets and therapeutic strategies for successful treatment of brain tumours in patients.

The neural stem cells (NSCs) are a small population of cells present in the subventricular zone that can proliferate, migrate and differentiate into neuro-glial cells in adult CNS (Mokry *et al*, 1996; Shihabuddin *et al*, 1999; Uchida *et al*, 2000). Although NSCs have unlimited potential to treat brain diseases (Groves *et al*, 1993; Lundberg *et al*, 1997; Rogister *et al*, 1999; Pluchino *et al*, 2003), it is believed that these resident stem cells are the potential source of brain tumours (Stupp and Hegi, 2007). The existence of cancer stem cells has been suggested for breast, prostate, colon and brain cancer. Failure to cure cancer has been attributed to the fact that typical therapies target rapidly proliferating tumour cells, which respond transiently, whereas sparing the tumour stem cells that has high tumorigenic potential (Bao *et al*, 2006). Recent studies have demonstrated the presence of CD133+ brain tumour stem cells (BTSCs) that has self-renewal, transplantation and metastasis properties in culture and in animal models (Singh *et al*, 2004). Brain tumour stem cells are considered responsible for the resistance and recurrence of brain tumours after radiation and chemotherapy in patients (Singh *et al*, 2004; Bao *et al*, 2006; Stupp and Hegi, 2007). However, there is no treatment available that can successfully target BTSCs in patients.

Nuclear receptors are a family of ligand-dependent transcription factors that mediate responses to steroids, retinoids, thyroid hormone and vitamin D and play key roles in development and regulation of inflammatory responses (Blumberg and Evans, 1998). Retinoic acid (RA) is a vitamin A derivative that activates RAR/RXR complex and induces neuro-glial differentiation of stem cells (Guan *et al*, 2001). Peroxisome proliferator-activated receptor (PPAR) is a member of the family of nuclear receptor transcription factors composed of three known subtypes PPAR*α*, PPAR*γ* and PPAR*δ* (Kliewer *et al*, 1992). Peroxisome proliferator-activated receptor gamma is expressed in many different tissues and regulates lipid metabolism, glucose homoeostasis, tumour progression and inflammation. Several fatty acids and ecosanoids function as physiological ligands for PPAR*γ*. The 15-deoxy Δ^12,14^-prostaglandin J2 (15d-PGJ2) is a natural ligand and thiazolidinediones, such as ciglitazone, are synthetic agonists for PPAR*γ* (Forman *et al*, 1995; Lehmann *et al*, 1995). Upon activation with specific ligands, PPAR*γ* heterodimerizes with RXR and induces gene expression associated with cell growth and differentiation. Peroxisome proliferator-activated receptor gamma agonists regulate adipogenesis and prevent obesity. Peroxisome proliferator-activated receptor gamma agonists also modulate glucose metabolism and insulin sensitivity, thereby reducing plasma glucose and insulin levels in type 2 diabetes (Schwartz *et al*, 1998). Peroxisome proliferator-activated receptor gamma agonists attenuate the clinical symptoms of colitis, arthritis, atherosclerosis, myocarditis, sepsis and multiple sclerosis in animal models (Kawahito *et al*, 2000; Claudel *et al*, 2001; Natarajan *et al*, 2003).

Interestingly, recent studies have shown that PPAR*γ* is expressed in normal and malignant human brain and that treatment with PPAR*γ* agonists induces growth arrest and apoptosis in brain tumour cells *in vitro* and in animal models *in vivo* (Strakova *et al*, 2004, 2005; Cellai *et al*, 2006; Grommes *et al*, 2006), but their effects on BTSCs are unknown. In this study, we show that PPAR*γ* agonists inhibit growth and expansion of CD133+ BTSCs as gliospheres in culture, further suggesting its use in the treatment of brain tumour.

## Materials and methods

### Reagents

The murine recombinant epidermal growth factor (EGF) and basic fibroblast growth factor (bFGF) were purchased from Chemicon International (Temecula, CA, USA). 15-Deoxy-Δ^12,14^-Prostaglandin J_2_ (15d-PGJ2) and ciglitazone were purchased from Calbiochem (La Jolla, CA, USA). Antibodies specific to Tyrosine kinase 2 (Tyk2), signal transduction and activator of transcription 3 (Stat3), PPAR*γ* and *β*-actin were purchased from Santa Cruz Biotechnology Inc. (Santa Cruz, CA, USA). The HRP-conjugated secondary Abs, all-*trans* retinoic acid (ATRA) and other chemicals were purchased from Sigma Chemicals Co. (St Louis, MO, USA). The anti-CD133 antibody conjugated with phycoerythrin (PE) and the isotype control were purchased from Miltenyi Biotec (Auburn, CA, USA). DeadEnd™ Fluorometric Tunel system was purchased from Promega, (Madison, WI, USA). Annexin-V-Fluos was purchased from Roche (Indianapolis, IN, USA).

### Cell culture

The U87MG and T98G brain tumour cell lines, established from human glioblastoma were obtained from American Type Culture Collection (ATCC, Manassas, VA, USA). The cells were cultured in Dulbecco's modified Eagle medium (DMEM) (Invitrogen, Carlsbad, CA, USA) supplemented with 10% FBS, 1 mM sodium pyruvate, 100 U ml^−1^ penicillin G, 100 *μ*g ml^−1^ streptomycin, 2 mM glutamine, 1 mM MEM non-essential amino acids and 50 *μ*M 2-mercaptoethanol in 5% CO_2_ incubator at 37°C. The cells were dissociated using 0.25% trypsin and 0.53 mM EDTA solution and subcultured once in 3–5 days.

### Gliosphere culture

To generate gliospheres, we adopted a culture condition standardized for neurospheres in our laboratory. Briefly, U87MG and T98G glioblastoma cells were dissociated from DMEM cultures using trypsin–EDTA solution and cultured in neurobasal medium (NBM) supplemented with B27 in the presence of 10 ng ml^−1^ bFGF and EGF. The cells (5 × 10^4^ per ml per well) were cultured in 12-well plates in 5% CO_2_ incubator at 37°C with a medium change every 2–3 days and photographed ( × 200) after 7–10 days using AX70 Olympus microscope. To test the effect of PPAR*γ* agonists on gliosphere formation, the cells were cultured in NBM with B27 and 10 ng ml^−1^ bFGF and EGF in the presence of 15d-PGJ2 and ATRA and photographed ( × 200) after 10 days. To test the reversibility of gliosphere formation, the cells were then cultured in fresh DMEM medium or NBM with B27 and 10 ng ml^−1^ bFGF and EGF in the absence of 15d-PGJ2 and ATRA for another 10 days. To determine the gliosphere counts, U87MG and T98G cells were cultured in 96-well tissue culture plates (5 × 10^3^ per 200 *μ*l per well) in NBM with B27 and 10 ng ml^−1^ EGF+bFGF in the presence of 0, 1, 2.5, 5 and 10 *μ*M ciglitazone, 15d-PGJ2 or ATRA for 7–10 days and the number of gliospheres counted under microscope.

### Proliferation assay

Proliferation of glioma and gliosphere cells was measured by ^3^H thymidine uptake assay. Briefly, U87MG and T98G glioma and gliosphere cells were cultured in 96-well tissue culture plates (1 × 10^4^ per 200 *μ*l per well) in NBM supplemented with B27 in the presence of 0, 1, 5 and 10 ng ml^−1^ EGF+bFGF in 5% CO_2_ incubator at 37°C. The cells were also cultured with 10 ng ml^−1^ EGF+bFGF in the presence of 15d-PGJ2, ciglitazone or ATRA. ^3^H thymidine (0.5 *μ*Ci ml^−1^) was added after 48 h, and the cells were dissociated and harvested after 72 h using a Tomtech harvester 96 (Hamden, CT, USA). The amount of ^3^H thymidine uptake was counted on Wallac Microbeta liquid scintillation counter (Perkin Elmer, Fremont, CA, USA) as a measure of proliferation.

### Flow cytometry

The U87MG and T98G glioma and gliosphere cells were cultured in 12-well tissue culture plates in NBM with B27 and 10 ng ml^−1^ EGF+bFGF in the presence of 5 *μ*M ciglitazone, 15d-PGJ2 or ATRA in 5% CO_2_ incubator at 37°C. After 72 h, the cells were harvested, dissociated and incubated in blocking buffer (1% BSA in PBS) at 4°C for 20 min. The cells were then stained with PE-conjugated anti-CD133 antibody or isotype control (1 : 10; Miltenyi Biotec) at 4°C for 1 h. The cells were washed three times with 0.1% BSA in PBS and analysed by a FACSort flow cytometry using CellQuest software (Becton Dickinson, San Jose, CA, USA).

### Cell cycle analysis

To determine the effect of PPAR*γ* agonists on cell cycle progression, U87MG and T98G gliospheres were cultured in NBM with B27 and 10 ng ml^−1^ EGF+bFGF in the presence of 5 *μ*M ciglitazone, 15d-PGJ2 or ATRA in 5% CO_2_ incubator at 37°C. After 24 h, the gliospheres were dissociated, fixed and permeabilized with 1% paraformaldehyde and 0.02% Triton X-100 in PBS at 4°C for 20 min. The cells were washed in PBS and stored in 70% ethanol at −20°C overnight. After washing in PBS, the cells were treated with 20 *μ*g ml^−1^ of RNase and stained with 50 *μ*g ml^−1^ of propidium iodide (Sigma Chemicals Co.) in PBS. The percentage of cells at different cell cycle stages (G0/G1, G2/M and S phase) was determined on the basis of DNA content by flow cytometry using ModFit LT2.0 software.

### Apoptosis assay

To determine the effect of PPAR*γ* agonists on apoptosis, U87MG and T98G gliospheres were cultured in neurobasal medium supplemented with B27 and 10 ng ml^−1^ EGF+bFGF in the presence of 5 *μ*M of 15d-PGJ2, ciglitazone or ATRA in 5% CO_2_ incubator at 37°C for 72 h. The cells were washed in PBS and stained with Annexin V-Flous in binding buffer (0.1 M HEPES/NaOH, pH 7.4, 1.4 M NaCl, 0.2 *μ*m sterile-filtered) containing propidium iodide according to the manufacturer's instruction (Roche). The cells were incubated at room temperature for 30 min in the dark and analysed by flow cytometry using Cell Quest Software. To further determine the extent of apoptosis, the cells were analysed by terminal deoxynucleotidyl transferase (TdT, TUNEL) assay according to the manufacturer's instruction (Promega, Madison, WI, USA). The cells were fixed in 1% paraformaldehyde for 20 min on ice, washed with PBS and stored overnight in 70% ethanol at −80°C. The samples were then washed with PBS, and incubated in equilibration buffer for 5 min and then in 50 *μ*l of TdT buffer at 37°C for 1 h. The cells were washed in PBS and analysed by flow cytometry. To examine DNA fragmentation, the cells were incubated overnight at 56°C in 300 *μ*l of digestion buffer (10 mM Tris-HCl, pH 8, 25 mM EDTA, pH 8, 0.5% SDS and 0.1 mg ml^−1^ proteinase K) and the genomic DNA was extracted using phenol/chloroform/isoamyl alcohol (25 : 24 : 1) followed by ammonium acetate/ethanol precipitation. The DNA samples were dissolved in TE buffer, resolved by 2% agarose gel electrophoresis and photographed under UV light.

### SDS–PAGE and western blot analysis

The U87MG and T98G gliosphere cells were stimulated with 0, 2, 5, 10 and 20 ng ml^−1^ EGF+bFGF in NBM+B27 at 37°C for 15 min. The cells were also pretreatmed with 5 and 15 *μ*M of ciglitazone, 15d-PGJ2 or ATRA at 37°C for 15 min and then stimulated with 10 ng ml^−1^ of EGF/bFGF for 15 min. The cells were washed in ice-cold PBS, and the whole cell lysates were prepared by boiling in lysis buffer (0.2 M Tris-HCl, pH 6.8, 0.8 *μ*g ml^−1^ SDS, 4% glycerol, 0.588 M *β*-mercaptoethanol, 0.05 M EDTA, 8 *μ*g ml^−1^ bromophenol blue) for 5 min. The whole cell lysates were resolved on 7.5% SDS−PAGE and transferred to nitrocellulose membrane using the gel electrophoresis and transfer system (Bio-Rad, Hercules, CA, USA). The residual binding sites in the membrane were blocked by incubation with TBST (10 mM Tris-HCl, pH 8.0, 150 mM NaCl and 0.05% Tween 20) containing 3% BSA for 1 h. The blots were washed three times in TBST at room temperature and incubated with anti-phospho-Tyk2 (1 : 1000), anti-phospho-Stat3 (1 : 2000) or anti-*β*-actin (1 : 5000) antibody in TBST containing 1% BSA at 4°C overnight. The membranes were then washed with TBST and incubated with peroxidase-conjugated anti-IgG antibody in TBST (1 : 10 000) for 1 h. The blots were developed by enhanced chemiluminescence (ECL) detection system and film (Amersham Life Sciences, Arlington Heights, IL, USA) according to the manufacturer's instruction. The gliospheres were also cultured in six-well plates in the presence of 5 *μ*M of 15d-PGJ_2_, ciglitazone, ATRA or DMSO control for 48 h and the cell lysates were analysed by western blot using anti-PPAR-*γ* antibody (1 : 1000) and peroxidase-conjugated anti-IgG antibody (1 : 10 000) for 1 h and visualised by ECL detection system.

### Quantitative reverse transcription PCR

The U87MG and T98G gliospheres were cultured in six-well plates (2 × 10^6^ cells per well) in NBM+B27 with 10 ng ml^−1^ EGF+bFGF in the presence of 5 *μ*M of 15d-PGJ_2_, ciglitazone or ATRA. After 36 h, the cells were harvested and the total RNA was extracted using TRIzol reagent (Invitrogen, Carlsbad, CA, USA) according to the manufacturer's instruction. The cDNA was reverse-transcribed by incubating 5 *μ*g of total RNA in 10 *μ*l of reaction mixture of random hexamer primers and master mix from TaqMan reverse transcription kit (Applied Biosystems, Branchburg, NJ, USA). For quantitative real-time PCR, 2 *μ*l of the cDNA (equivalent to 0.1 *μ*g total RNA) was amplified in TaqMan Universal Master Mix with optimised concentrations of PPAR*γ* primer sets and probes in a fast optical 96-well reaction plate using the 7900 Fast Sequence Detection Real-time PCR System (Applied Biosystems, Foster City, CA). The results were analysed using the Prism 7900 relative quantification (ΔΔ*C*_t_) study software (Applied Biosystems, Foster City, CA). The level of PPAR*γ* was normalised to GAPDH and expressed as arbitrary fold change compared with control sample.

### Statistical analysis

The experiments were repeated three or more times and the data were analysed by ANOVA (Graphpad Prism 5.0). The *P*-values less than 0.05 were considered significant.

## Results

### PPAR*γ* agonists inhibit EGF+bFGF-induced gliosphere formation in culture

**Figure 1:**
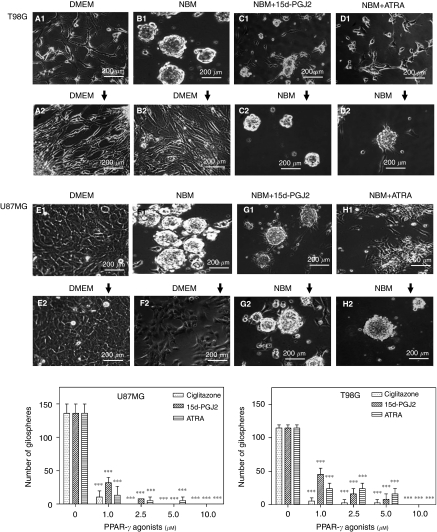
Inhibition of gliosphere formation by PPAR*γ* agonists. The T98G and U87MG human glioblastoma cells were cultured in DMEM medium with 10% FBS (**A1** and **E1**) or NBM with B27 and 10 ng ml^−1^ EGF+bFGF in the absence (**B1** and **F1**) or presence of 1.0 *μ*M 15d-PGJ2 (**C1** and **G1**) or ATRA (**D1** and **H1**) and photographed ( × 200) after 10 days. The cells were then cultured in fresh DMEM medium with 10% FBS (**A2**, **B2** and **E2**, **F2**) or NBM with B27 and 10 ng ml^−1^ EGF+bFGF in the absence of 15d-PGJ2 (**C2** and **G2**) or ATRA (**D2** and **H2**) and photographed ( × 200) after 10 days. The T98G and U87MG cells were also cultured in NBM with B27 and EGF+bFGF in the absence or presence of Ciglitazone, 15d-PGJ2 and ATRA, the number of gliospheres counted after 10 days and the mean±s.e.m. of triplicates presented in the histogram. The *P*-values are shown as (^*^P<0.05), (^**^P<0.01) and (^***^P<0.001) and the figure is a representative for three independent experiments.

To study the effect of PPAR*γ* agonists on the expansion of BTSCs from glioma, we first established culture conditions to generate gliospheres *in vitro*. As shown in Figure 1, *in vitro* culture of T98G and U87MG human glioma cells grow as monolayer in DMEM medium with 10% FBS (a1, e1). But *in vitro* culture of glioma cells in NBM+B27 with EGF+bFGF promotes gliosphere formation in 5–7 days that increase in size by 7–10 days (b1, f1). Interestingly, *in vitro* treatment with PPAR*γ* agonist 15d-PGJ2 or ATRA resulted in a significant decrease in the number and size of gliospheres in T98G (c1, d1) and U87MG cells (g1, h1), suggesting that PPAR*γ* agonists inhibit gliosphere formation in culture. The U87MG and T98G gliospheres grow as monolayer after subculture in DMEM with 10% FBS (b2, f2). On the other hand, subculture of 15d-PGJ2- or ATRA-treated T98G cells (c2, d2) and U87MG cells (g2, h2) in fresh NBM with EGF+bFGF in the absence of nuclear receptor agonists resulted in gliosphere formation in 7–10 days. Further analyses showed that *in vitro* treatment with 15d-PGJ2, ciglitazone or ATRA resulted in a dose-dependent decrease in the number of gliospheres generated from T98G and U87MG cells following culture in NBM with B27 and EGF+FGF. These results suggest that PPAR*γ* agonists inhibit EGF+bFGF-induced gliosphere formation in culture.

### PPAR*γ* agonists inhibit EGF+bFGF-induced expansion of CD133+ gliosphere cells in culture

**Figure 2:**
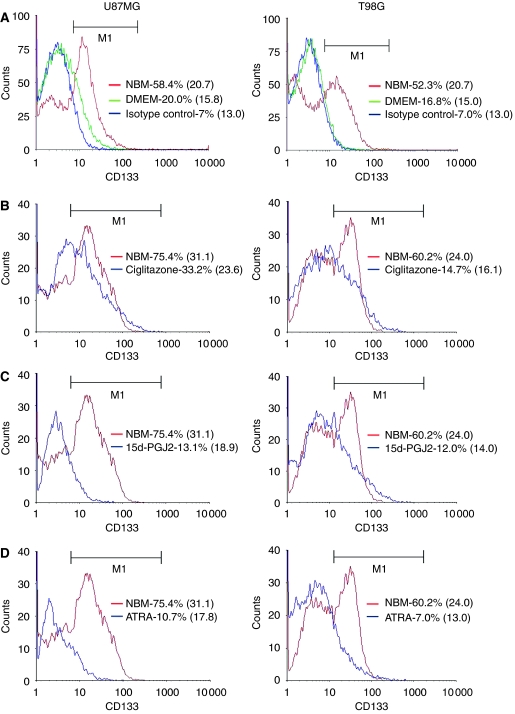
Inhibition of CD133+ BTSC expansion by PPAR*γ* agonists. The U87MG and T98G-sphere cells were cultured in NBM+B27 with EGF+FGF in the absence (**A**) or presence of 1 *μ*M ciglitazone (**B**), 15d-PGJ2 (**C**) or ATRA (**D**). After 5 days, the cells were stained with anti-CD133-PE or isotype-matched control antibody and analysed by flow cytometer. The percentage of CD133+ cells and mean fluorescence intensity in brakets are shown.

As CD133 has been used as a marker to identify BTSCs, we analysed the presence of CD133-positive cells in gliospheres generated in culture. As shown in Figure 2, U87MG and T98G cells show very low levels of CD133+ BTSCs, which increased significantly in gliospheres. However, the U87MG and T98G cells cultured in DMEM showed 20 and 16.8% CD133+ cells with a mean fluorescence intensity (MFI) of 16 and 15 that increased to 58 and 52% with an MFI of 21 and 20, respectively, following culture as gliospheres (Figure 2A). Interestingly, *in vitro* treatment with PPAR*γ* agonists resulted in a significant decrease in the number of CD133+ cells in gliospheres. In this experiment, however, the U87MG-spheres showed 75% CD133+ BTSCs with an MFI of 31 that decreased to 33, 13 and 11% with an MFI of 24, 19 and 18 following treatment with ciglitazone, 15d-PGJ2 or ATRA, respectively (Figure 2B–D). Similarly, T98G-gliospheres showed 60% CD133+ BTSCs with an MFI of 24 that decreased to 15, 12 and 7% with an MFI of 16, 14 and 13 following treatment with ciglitazone, 15d-PGJ2 or ATRA, respectively (Figure 2B–D). These results suggest that PPAR*γ* agonists and retinoic acid inhibit the expansion of CD133+ BTSCs in culture.

### PPAR*γ* agonists inhibit EGF+bFGF-induced proliferation of gliosphere cells

**Figure 3:**
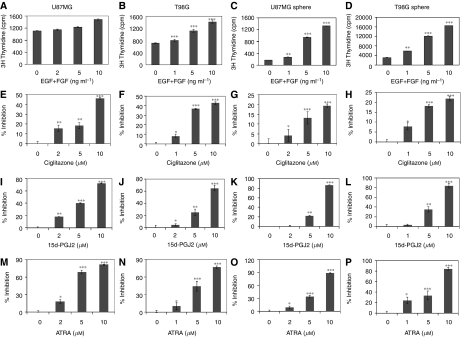
Inhibition of BTSC proliferation by PPAR*γ* agonists. The U87MG and T98G cells (**A**, **B**) and the gliosphere cells (**C**, **D**) were cultured in NBM+B27 with different concentrations of EGF+FGF. The U87MG and T98G cells were cultured in NBM with B2 and 10 ng ml^−1^ bFGF+EGF in the presence of ciglitazone (**E**, **F**), 15d-PGJ2 (**I**, **J**) or ATRA (**M**, **N**) in 96-well tissue culture plates in triplicate. U87MG- and T98G-gliosphere cells were also cultured in NBM+B27 with 10 ng ml^−1^ EGF+FGF in the presence of ciglitazone (**G**, **H**), 15d-PGJ2 (**K**, **L**) or ATRA (**O**, **P**). After 48 h, ^3^H thymidine (0.5 *μ*Ci per well) was added, and the amount of ^3^H thymidine uptake was counted on a Perkin Elmer Microbetaplate liquid scintillation counter as a measure of proliferation. The values are mean of triplicate (±s.e.m.) with *P*-values shown as (^*^P<0.05), (^**^P<0.01) and (^***^P<0.001). The figure is representative of three independent experiments.

To test the anti-tumour activity of PPAR*γ* agonists on BTSCs, we measured cell proliferation in culture. As shown in Figure 3, *in vitro* culture of U87MG (a), T98G (b), U87MG-gliosphere (c) and T98G-gliosphere (d) cells in NBM+B27 with EGF+bFGF induced a dose-dependent increase in proliferation, with a significantly higher response in gliosphere cells. Interestingly, PPAR*γ* agonists induced a strong dose-dependent inhibition of proliferation in both glioma and gliosphere cells. *In vitro* culture of U87MG-gliosphere cells in NBM+B27 with EGF+FGF in the presence of ciglitazone (g), 15d-PGJ2 (k) or ATRA (o) resulted in 21, 86 and 92% inhibition of proliferation, respectively. Similarly, T98G-gliosphere cells cultured in NBM+B27 with EGF+FGF in the presence of ciglitazone (h), 15d-PGJ2 (l) or ATRA (p) resulted in 23, 84 and 86% inhibition of proliferation, respectively. Moreover, *in vitro* treatment of U87MG glioma cells with ciglitazone (e), 15d-PGJ2 (i) or ATRA (m) cultured in NBM+B27 with EGF+FGF resulted in 46, 72.26 and 81% inhibition of proliferation, respectively. Similarly, *in vitro* treatment of T98G glioma cells with ciglitazone (f), 15d-PGJ2 (j) or ATRA (n) cultured in NBM+B27 with EGF+FGF resulted in 44, 65 and 78% inhibition of proliferation, respectively. These results suggest that PPAR*γ* agonists inhibit proliferation of BTSCs in culture.

### PPAR*γ* agonists induce cell cycle arrest in gliosphere cells

**Figure 4:**
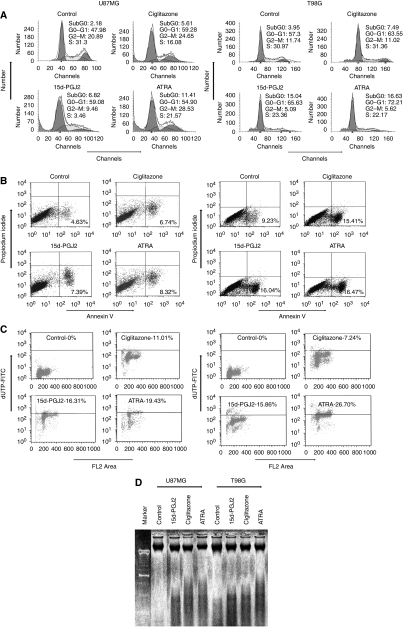
Induction of cell cycle arrest and apoptosis by PPAR*γ* agonists in BTSCs. U87MG and T98G-sphere cells (2 × 10^6^ cells) were cultured in NBM with B27 and EGF+FGF in the presence of 1 *μ*M ciglitazone, 15d-PGJ2 or ATRA. The cells were harvested after 48 h and stained with propidium iodide (**A**), Annexin V-FITC (**B**) or dUTP-FITC (**C**) and analysed by flow cytometry. Genomic DNA was extracted and analysed by agarose gel electrophoresis to visualise DNA fragmentation (**D**). The figure is representative of three independent experiments.

We then examined whether the inhibition of proliferation by PPAR*γ* agonists was due to cell cycle arrest or apoptosis in gliospheres. As shown in Figure 4A, treatment of T98G and U87MG-derived gliosphere cells with ciglitazone, 15d-PGJ2 or ATRA resulted in cell cycle arrest at G0/G1 phase. U87MG-sphere cells cultured in NBM+B27 with EGF+bFGF showed 48% G0/G1 cells, 21% G2/M cells and 31% S-phase cells; however, addition of 5 *μ*M ciglitazone, 15d-PGJ2 or ATRA resulted in 59, 59 and 55% G0/G1 cells, 25, 10 and 24% G2/M cells and 16, 32 and 22% S-phase cells in 24 h. Similarly, T98G-sphere cells cultured in NBM+B27 with EGF+bFGF showed 57% G0/G1 cells, 12% G2/M cells and 31% S-phase cells, and the addition of 5 *μ*M ciglitazone, 15d-PGJ2 or ATRA resulted in 64, 66 and 72% G0/G1 cells, 11, 5 and 6% G2/M cells and 32, 23 and 22% S-phase cells in 24 h. These results suggest that PPAR*γ* agonists regulate cell cycle progression of BTSCs in culture.

### PPAR*γ* agonists induce apoptosis in gliosphere cells

We then examined whether PPAR*γ* agonists induce apoptosis in BTSCs. As shown in Figure 4B, U87MG- and T98G-gliosphere cells cultured in NBM+B27 with EGF+bFGF showed low apoptotic cell death. However, treatment with PPAR*γ* agonists resulted in a significant increase in apoptotic cells as measured by Annexin V staining. U87MG-gliosphere cells cultured in NBM+B27 with EGF+bFGF showed 5% apoptosis that increased to 7, 7 and 8% following the addition of 5 *μ*M ciglitazone, 15d-PGJ2 or ATRA, respectively. Similarly, T98G-sphere cells cultured in NBM+B27 with EGF+bFGF showed 9% apoptosis that increased to 15, 16 and 17% following the addition of 5 *μ*M ciglitazone, 15d-PGJ2 or ATRA, respectively. To further confirm the results, we performed Tunel assay in gliosphere cells following treatment with PPAR*γ* agonists. As shown in Figure 4C, U87MG-sphere cells cultured in NBM+B27 with EGF+bFGF showed 0% apoptosis that increased to 11, 16 and 19% following the addition of 5 *μ*M ciglitazone, 15d-PGJ2 or ATRA, respectively. Similarly, T98G-sphere cells cultured in NBM+B27 with EGF+bFGF showed 0% apoptosis that increased to 7, 16 and 26% following the addition of 5 *μ*M ciglitazone, 15d-PGJ2 or ATRA, respectively. Further analysis by agarose gel electrophoresis showed that U87MG and T98G-gliosphere cells treated with 5 *μ*M ciglitazone, 15d-PGJ2 or ATRA, but not in control samples, showed the evidence of DNA fragmentation (Figure 4D). These results suggest that PPAR*γ* agonists induce modest apoptosis of BTSCs in culture.

### PPAR*γ* agonists inhibit EGF/bFGF-induced Jak-Stat pathway in gliosphere cells

**Figure 5:**
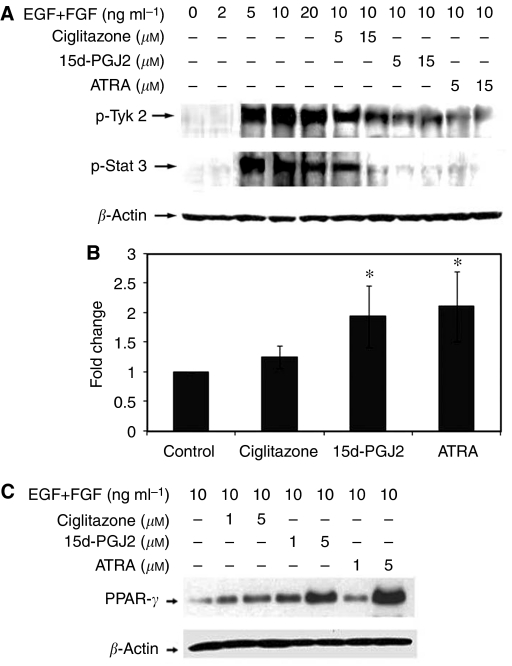
Inhibition of EGF/FGF signalling and induction of PPAR*γ* expression by PPAR*γ* agonists in BTSCs. T98G-sphere cells (2 × 10^6^ cells) were cultured in NBM with B27 and EGF+FGF in the absence or presence of ciglitazone, 15d-PGJ2 or ATRA. The cells were harvested after 15 min and the whole cell lysates were analysed by SDS–PAGE and western blot using phosphospecific Tyk2 or Stat3 antibodies and visualised by ECL detection system (**A**). The cells were harvested after 36 h and total RNA was analysed for the expression of PPAR*γ* by real-time RT–PCR using GAPDH as internal control (**B**). The values are mean of triplicate (±s.e.m.) and *P*-values shown as (^*^P<0.05) in the figure. The cells were harvested after 48 h and total protein was analysed for the expression of PPAR*γ* analysed by SDS–PAGE and western blot using anti-PPAR*γ* antibodies and visualised by ECL detection system (**C**). The figure is representative of three independent experiments.

To study the mechanisms in the regulation of BTSCs by PPAR*γ* agonists, we examined their effects on EGF+bFGF-induced activation of Jak-Stat signalling pathway. As shown in Figure 5A, *in vitro* stimulation of T98G-sphere cells with EGF+bFGF induced the tyrosine phosphorylation of Tyk2 and Stat3 in 15 min in a dose-dependent manner. Interestingly, pretreatment with 15d-PGJ2, ciglitazone or ATRA for 15 min resulted in a dose-dependent decrease in the tyrosine phosphorylation of Tyk2 and Stat3 in T98G-sphere cells. Whereas 5 *μ*M agonists induced partial inhibition, treatment with 15 *μ*M agonists resulted in almost complete inhibition of Tyk2 and Stat3 phosphorylation in gliosphere cells. These results suggest that PPAR*γ* agonists inhibit the expansion of BTSCs by targeting EGF+FGF-induced Tyk2-Stat3 signalling pathway.

### PPAR*γ* agonists induce the expression of PPAR*γ* in gliosphere cells

To further understand the mechanisms in the regulation of BTSCs by PPAR*γ* agonists, we examined the expression of PPAR*γ* and its regulation by agonists in gliosphere cells. As shown in Figure 5B, real-time PCR analysis showed that T98G-sphere cells cultured in NBM+B27 with EGF+bFGF showed detectible levels of PPAR*γ* mRNA expression that increased significantly following treatment with 5 *μ*M ciglitazone, 15d-PGJ2 or ATRA for 36 h in culture. Western blot analysis showed that T98G-sphere cells cultured in NBM+B27 with EGF+bFGF showed detectible levels of PPAR*γ* protein that increased significantly following treatment with 5 *μ*M ciglitazone, 15d-PGJ2 or ATRA for 48 h in culture. These results suggest that BTSCs express functionally active PPAR*γ*, and the expression levels are increased following treatment with PPAR*γ* agonists in culture.

## Discussion

The identification of tumour stem cells has revolutionized the basic understanding of the biology, drug discovery and treatment of cancer. Although CD133 has been commonly used as a marker to identify BTSCs, many tumours often present a small population of CD133+ cells, making it difficult to isolate sufficient cells for drug discovery. In this study, we approached to expand the BTSC population as gliospheres from U87MG and T98G human glioblastoma cells according to a protocol standardized in our laboratory. We found that *in vitro* culture of human glioblastoma cells with EGF+bFGF induced the generation of gliospheres with more than 50% of cells positive for CD133, suggesting the successful expansion of BTSCs in culture. Interestingly, treatment with PPAR*γ* agonists resulted in a reversible inhibition of gliosphere formation in culture. Peroxisome proliferator-activated receptor gamma agonists also inhibited the proliferation of BTSCs by inducing cell cycle arrest and apoptosis. The induction of growth arrest and apoptosis by PPAR*γ* agonists was associated with the expression of PPAR*γ* and inhibition of Tyk2-Stat3 signalling pathway in BTSCs, further suggesting the use of PPAR*γ* agonists in the treatment of brain tumours.

Epidermal growth factor and FGF are two important growth factors that induce the expansion of BTSCs as gliospheres in culture. Epidermal growth factor receptor (EGFR) is a 170 kDa transmembrane tyrosine kinase that binds to EGF (Wells, 1999; Johnston *et al*, 2006) and mediates signalling pathways leading to survival, proliferation, migration and invasion of glioma cells (Wells, 1999). The expression of EGFR is often amplified in human glioma, but is undetectable or weakly expressed in normal brain (Martens *et al*, 2008). Fibroblast growth factor-2 (bFGF) is a ubiquitously expressed prototype of the family of 20 proteins (Szebenyi and Fallon, 1999) with mitogenic, angiogenic, neurotrophic and oncogenic activities (Zagzag *et al*, 1990). Fibroblast growth factor-2 plays a critical role in nervous system development (Eckenstein, 1994), and dysregulated expression is implicated in the pathogenesis of brain tumours. FGF-2 is upregulated during reactive gliosis (Finklestein *et al*, 1988; Frautschy *et al*, 1991) and overexpressed in malignant gliomas (Murphy *et al*, 1989; Morrison *et al*, 1994). The expression level of FGF-2 correlates with tumour grade, extent of anaplasia and clinical outcomes in glioma (Takahashi *et al*, 1992; Bredel *et al*, 1997). Thus EGF and FGF receptors are now considered as prime targets for the specific delivery of a variety of diagnostic and therapeutic agents (Johnston *et al*, 2006; Baguma-Nibasheka *et al*, 2007; Fukushima *et al*, 2008). However, the role of EGF and bFGF and their receptors on growth and expansion of BTSCs are not well defined. In this study, we showed that EGF+bFGF induce the expansion of CD133+ BTSCs as gliospheres in culture. Recent studies have shown that tumour environment dictates cancer stem cell expression and invasive phenotype *in vivo* (Annabi *et al*, 2008). Although the exact mechanisms are not known, it is most likely that the growth factors induce the expansion of CD133+ BTSCs or de-differentiation of glioblastoma cells to CD133+ BTSCs in culture (Kondo, 2007). Earlier studies have shown that PPAR*γ* agonists induce growth arrest and apoptosis in glioblastoma cells in culture (Liu *et al*, 2004; Morosetti *et al*, 2004). In this study, we show for the first time that PPAR*γ* agonists inhibit the generation of gliospheres and expansion of CD133+ BTSCs in culture.

Drugs that reduce cell proliferation and survival has high therapeutic potential in human tumours. Earlier studies have shown that PPAR*γ* agonists induced growth arrest and apoptosis in glioblastoma cells without affecting primary astrocytes (Zander *et al*, 2002), demonstrating its anti-neoplastic potency in humans. In this study, we found that PPAR*γ* agonists induce a strong and dose-dependent inhibition of proliferation in gliosphere cells in culture. The PPAR*γ* agonists also induced a modest cell cycle arrest and apoptosis in gliosphere cells, suggesting their anti-tumour activity in BTSCs. The exact mechanisms how PPAR*γ* agonists induce growth arrest and apoptosis in BTSCs are not known. Signalling through its receptor tyrosine kinase, EGF and bFGF induce the activation of Jak-Stat pathway leading to proliferation and survival of different cell types (Raz *et al*, 1999; Burdon *et al*, 2002). We have shown earlier that PPAR*γ* agonists inhibit cytokine-induced activation of Jak-Stat pathway in immune cells and LIF-induced activation of Jak-Stat pathway in mouse embryonic stem cells (Rajasingh and Bright, 2006). In this study, we found that EGF+bFGF induces tyrosine phosphorylation of Stat3 and Tyk2, and PPAR*γ* agonists inhibit tyrosine phosphorylation of Stat3 and Tyk2 in both U87MG and T98G-gliosphere cells. The inhibition of Stat3 proteins by PPAR*γ* agonists may be due to the inhibition of upstream Tyk2 or its direct effect on Stat3 protein. Peroxisome proliferator-activated receptor gamma agonists can also regulate Tyk2-Stat3 pathway by activating the negative regulators, such as suppressor of cytokine signalling or SHP-1 proteins in BTSCs (Park *et al*, 2003). Although the possible inhibition of other growth signalling pathways by PPAR*γ* agonists cannot be ruled out, our results suggest that the inhibition of Jak-Stat pathway be an important mechanism by which PPAR*γ* agonists induces growth arrest and apoptosis in BTSCs.

Earlier studies have shown that glioma cells express PPAR*γ* and that the levels of expression can be modulated by specific agonists (Morosetti *et al*, 2004). In this study, we showed that the gliosphere cells express detectible levels of PPAR*γ* mRNA and protein and that treatment with PPAR*γ* agonists increased the expression, suggesting its functional significance to BTSCs and the involvement of PPAR*γ*-dependent mechanisms in the induction of growth arrest and apoptosis by PPAR*γ* agonists in BTSCs. Although the precise molecular basis of the antineoplastic mechanisms of PPAR*γ* agonists is not fully understood, our findings suggest that the clinically used PPAR*γ* agonists may offer new therapies to target BTSCs for the treatment of brain tumours in patients.
